# Supplementary material for: Intervene: a tool for intersection and visualization of multiple gene or genomic region sets
Source: BMC Bioinformatics. 2017 May 31;18:287. doi: 10.1186/s12859-017-1708-7 (PMC5452382; doi:10.1186/s12859-017-1708-7)
Supplement: Supplementary file 1 — A PDF version of detail documentation including installation instruction and how to use the command line interface and web application (PDF 1429 kb) [file 12859_2017_1708_MOESM1_ESM.pdf]

Intervene Documentation

Release v0.5.8

Aziz Khan

Mar 25, 2017

Table of contents

1 Introduction **3**

2 Installation **5**
2.1 Prerequisites 5
2.2 Install Intervene 6

3 How to use Intervene **9**
3.1 Run Intervene on test data 10

4 Intervene modules **11**
4.1 Venn diagram module 11
4.2 UpSet plot module 12
4.3 Pairwise intersection module 13

5 Example gallery **15**
5.1 Venn module examples 15
5.2 UpSet module examples 16
5.3 Pairwise module examples 16

6 Interactive Shiny App **21**
6.1 Introduction 21
6.2 Venn module 21
6.3 UpSet module 21
6.4 Pairwise module 23
6.5 Availability 24

7 Support **25**

8 Citation **27**

Welcome to Intervene - a tool for intersection and visualization of multiple genomic region sets

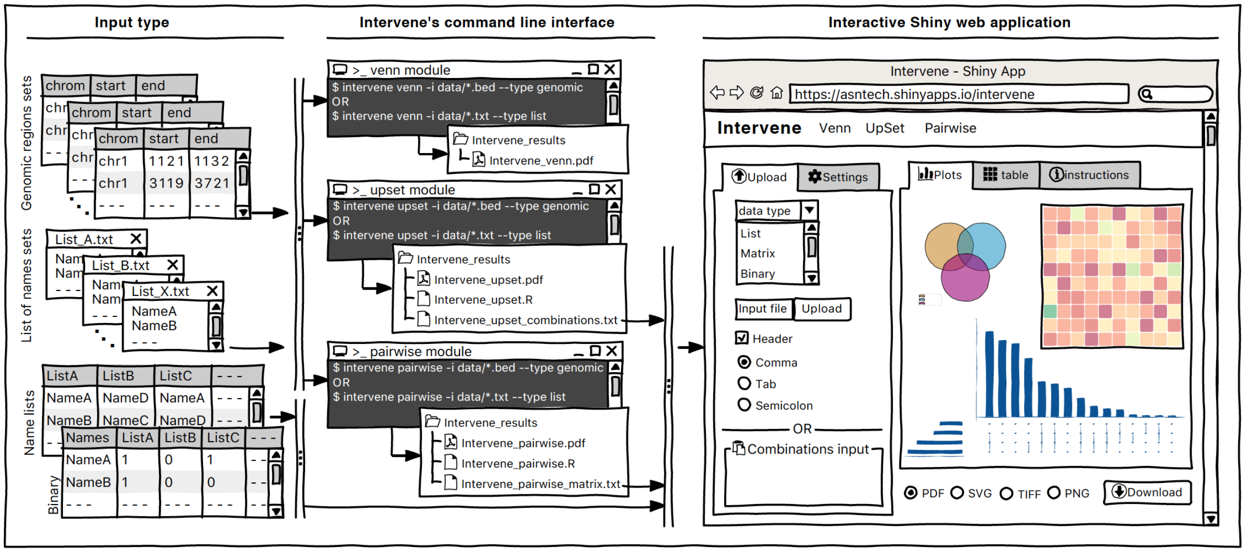

CHAPTER 1

Introduction

Intervene is a tool for intersection and visualization of multiple genomic region and gene sets (or lists of items).

Intervene provides an easy and automated interface for effective intersection and visualization of genomic region sets or lists of items, thus facilitating their analysis and interpretations. Intervene contains three modules.

- *venn* to compute Venn diagrams of up-to 6 sets
- *upset* to compute UpSet plots of multiple sets
- *pairwise* to compute and visualize intersections of genomic sets as clustered heatmap.

Intervene gives user flexibility to choose figure colors, labels, size, quality, and type to make them as publication standard.

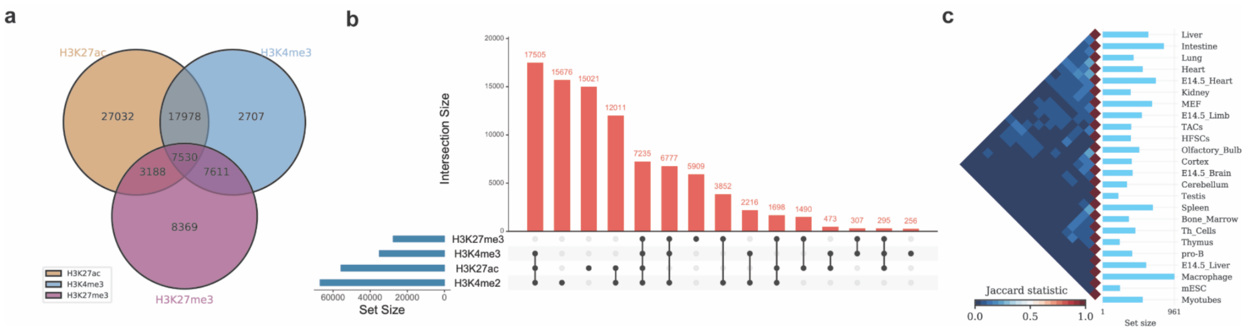

CHAPTER 2

Installation

Prerequisites

Intervene requires the following Python modules and R packages:

- Python (=> 2.7): https://www.python.org/
- BEDTools (Latest version): https://github.com/arq5x/bedtools2
- pybedtools (>= 0.7.9): https://daler.github.io/pybedtools/
- Pandas (>= 0.16.0): http://pandas.pydata.org/
- Seaborn (>= 0.7.1): http://seaborn.pydata.org/
- R (>= 3.0): https://www.r-project.org/
- R packages including UpSetR, corrplot

Install BEDTools

Intervene is using pybedtools, which is a Python wrapper for the BEDTools. BEDTools should be installed before using Intervene. It is recomended to have the latest version of the tool. Please read the installation instructions at https://github.com/arq5x/bedtools2 to install BEDTools, and make sure it is accessible through your PATH variable.

Install required Python modules

Intervene takes care of the installation of all the required Python modules. If you already have a working installation of Python, the easiest way to install the required Python modules is by installing Intervene using `pip`. If you're setting up Python for the first time, we recommend to install it using the Anaconda Python distribution http://continuum.io/downloads. These come with several helpful scientific and data processing libraries. These are available for platforms including Windows, Mac OSX and Linux.

If you want to install the required Python modules manually, you can use the following commands.

Install pybedtools

Install it from PyPi

```
pip install pybedtools
```

or using conda

```
conda install -c bioconda pybedtools
```

Read more details about ''pybedtools" installation: https://daler.github.io/pybedtools/main.html

Install Pandas

Install it from PyPi

```
pip install pandas
```

Or install with conda

```
conda install pandas
```

Install required R packages

Intervene rquires three R packages, UpSetR , corrplot for visualization and Cairo to generate high-quality vector and bitmap figures. To install these, open R/RStudio and use the following command.

```
install.packages(c("UpSetR", "corrplot","Cairo"))
```

Install Intervene

You can install a stable version of Intervene by using `pip` from PyPi or a development version by using `git` from our bitbucket repository at https://bitbucket.org/CBGR/intervene.

Install using *pip*

You can install Intervene either from PyPi using pip or install it from the source. Please make sure you have already installed the above mentioned python libraries required to run Intervene.

Install from PyPi:

```
pip install intervene
```

Install development version from *Bitbucket*

If you have *git* installed, use this:

```
git clone https://bitbucket.org/CBGR/intervene.git
cd intervene
python setup.py sdist install
```

Install development version from *GitHub*

If you have *git* installed, use this:

```
git clone https://github.com/asntech/intervene.git
cd intervene
python setup.py sdist install
```

CHAPTER 3

How to use Intervene

Once you have installed Intervene, you can type:

```
intervene --help
```

This will show the main help, which lists the three subcommands/modules: `venn`, `upset`, and `pairwise`.

```
usage: intervene <subcommand> [options]

positional arguments <subcommand>:
  {venn,upset,pairwise}
                        List of subcommands
    venn                Venn diagram of intersection of genomic regions or list sets␣
↪(upto 6-way).
    upset               UpSet diagram of intersection of genomic regions or list sets.
    pairwise            Pairwise intersection and heatmap of N genomic region sets in
↪<BED/GTF/GFF> format.

optional arguments:
  -h, --help            show this help message and exit
  -v, --version         show program's version number and exit
```

To view the help for the individual subcommands, please type:

To view `venn` module help, type

```
intervene venn --help
```

To view `upset` module help, type

```
intervene upset --help
```

To view `pairwise` module help, type

```
intervene pairwise --help
```

Run Intervene on test data

To run Intervene using example data, use the following commands. To access the test data make sure you have `sudo` or `root` access.

To run `venn` module with test data, type

```
intervene venn --test
```

To run `upset` module with test data, type

```
intervene upset --test
```

To run `pairwise` module with test data, type

```
intervene pairwise --test
```

If you have installed Intervene locally from the source code, you may have problem to find test data. You can download the test data here https://github.com/asntech/intervene/tree/master/intervene/example_data and point to it using `-i` instead of `--test`.

```
./intervene/intervene venn -i intervene/example_data/ENCODE_hESC/*.bed
./intervene/intervene upset -i intervene/example_data/ENCODE_hESC/*.bed
./intervene/intervene pairwise -i intervene/example_data/dbSUPER_mm9/*.bed
```

These subcommands will save the results in the current working directory with a folder named `Intervene_results`. If you wish to save the results in a specific folder, you can type:

```
intervene <module_name> --test --output ~/path/to/your/results/folder
```

CHAPTER 4

Intervene modules

Intervene provides three types of plots to visualize intersections of genomic regions and list sets. These are pairwise heatmap of N genomic region sets, classic Venn diagrams of genomic regions and list sets of up to 6-way and UpSet plots.

Venn diagram module

Once you have installed Intervene, you can type:

Usage:

```
intervene venn [options]
```

Note: Please scroll down to see a detailed summary of available **options**.

Help:

```
intervene venn --help
```

Example:

```
intervene venn -i path/to/BED/files/*.bed
```

This will save the results in the current working directory with a folder named `Intervene_results`. If you wish to save the results in a specific folder, you can type:

```
intervene venn -i path/to/BED/files/*.bed --output ~/results/path
```

Summary of options

Option	Description
-h, –help	To show the help message and exit
-i	Input genomic regions in (BED/GTF/GFF) format or lists of genes/SNPs IDs. For files in a directory use *.<extension>. e.g. *.bed
–type	{genomic,list}. Type of input sets. Genomic regions or lists of genes/SNPs. Default is `genomic`
–names	Comma-separated list of names as labels for input files. If it is not set file names will be used as labels. For example: –names=A,B,C,D,E,F
–filenames	Use file names as labels instead. Default is `False`
–colors	Comma-separated list of matplotlib-valid colors. E.g., –colors=r,b,k
-o, –output	Output folder path where results will be stored. Default is current working directory.
–figtype	{pdf,svg,ps,tiff,png} Figure type for the plot. e.g. –figtype svg. Default is `pdf`
–figsize	Figure size as width and height.e.g. –figsize 12 12.
–fontsize	Font size for the plot labels. Default is `14`
–dpi	Dots-per-inch (DPI) for the output. Default is: `300`
–fill	{number,percentage} Report number or percentage of overlaps (Only if –type=list). Default is `number`
–test	This will run the program on test data.

UpSet plot module

Once you have installed Intervene, you can type:

Usage:

```
intervene upset [options]
```

Note: Please scroll down to see a detailed summary of available **options**.

Help: You can also see list of options by typing this on the terminal.

```
intervene upset --help
```

Example:

```
intervene upset -i path/to/BED/files/*.bed
```

This will save the results in the current working directory with a folder named `Intervene_results`. If you wish to save the results in a specific folder, you can type:

```
intervene upset -i path/to/BED/files/*.bed --output ~/results/path
```

Summary of options

Option	Description
-h, –help	show this help message and exit
-i, –input	Input genomic regions in <BED/GTF/GFF/VCF> format or list files. For files in a directory use *.<ext>. e.g. *.bed
–type	Type of input sets. Genomic regions or lists of genes sets {genomic,list}. Default is `genomic`
–names	Comma-separated list of names as labels for input files. If it is not set file names will be used as labels. For example: –names=A,B,C,D,E,F
–filenames	Use file names as labels instead. Default is `True`
-o, –output	Output folder path where plots will store. Default is current working directory.
–order	The order of intersections of sets {freq,degree}. e.g. –order degree. Default is `freq`
–ninter	Number of top intersections to plot. Default is `30`
–showzero	Show empty overlap combinations. Default is `False`
–showsize	Show intersection sizes above bars. Default is `True`
–mbcolor	Color of the main bar plot. Default is `gray23`
–sbcolor	Color of set size bar plot. Default is `#56B4E9`
–mblabel	The y-axis label of the intersection size bars. Default is `No of Intersections`
–sxlabel	The x-axis label of the set size bars. Default is `Set size`
–figtype	Figure type for the plot. e.g. –figtype svg {pdf,svg,ps,tiff,png} Default is `pdf`
–figsize	Figure size for the output plot (width,height).
–dpi	Dots-per-inch (DPI) for the output. Default is `300`
–scriptonly	Set to generate Rscript only, if R/UpSetR package is not installed. Default is `False`
–showshiny	Print the combinations of intersections to input to Shiny App. Default is `False`

Pairwise intersection module

Once you have installed Intervene, you can type:

Usage:

```
intervene pairwise [options]
```

Note: Please scroll down to see a detailed summary of available **options**.

Help:

```
intervene pairwise --help
```

Example:

```
intervene pairwise -i path/to/BED/files/*.bed --type jaccard --htype tribar
```

This will save the results in the current working directory with a folder named `Intervene_results`. If you wish to save the results in a specific folder, you can type:

```
intervene pairwise -i path/to/BED/files/*.bed --type jaccard --htype tribar --output ~
↪/results/path
```

Summary of options

Option	Description
-h, –help	show this help message and exit
-i	Input genomic regions in (BED/GTF/GFF) format. For files in a directory use *.<extension>. e.g. *.bed
–type	{genomic,list}. Type of input sets. Genomic regions or lists of genes/SNPs. Default is `genomic`
–compute	Compute count/fraction of overlaps or statistical relationships. {`count`, `frac`, `jaccard`, `fisher`, `reldist`}
	–compute=count - calculates the number of overlaps.
	–compute=frac - calculates the fraction of overlap.
	–compute=jaccard - calculate the Jaccard statistic. Read more details here
	–compute=reldist - calculate the distribution of relative distances. Read more details here
	–compute=fisher - calculate Fisher‘s statistic. Read more details here
	Note: For jaccard and reldist regions should be pre-shorted or set –sort‘‘
–corr	Compute the correlation. By default set to False
–corrtype	Select the type of correlation from `pearson`, `kendall` or `spearman`.
	–corrtype=pearson: computes the Pearson correlation. (Default)
	–corrtype=kendall: computes the Kendall correlation.
	–corrtype=spearman: computes the Spearman correlation.
	Note: This only works if –corr is set.
–htype	{tribar,color,pie,circle,square,ellipse,number,shade}. Heatmap plot type. Default is `tribar`.
	Read the below note for `tribar` option.
–triangle	Show lower/upper triangle of the matrix as heatmap. Default is `lower`
–diagonal	Show the diagonal values in the heatmap. Default is `False`.
–names	Comma-separated list of names as labels for input files. If it is not set file names will be used as labels. For example: –[illegible]
–filenames	Use file names as labels instead. Default is `False`.
–sort	Set this only if your files are not sorted. Default is `False`.
–genome	Required argument if –compute=fisher. Needs to be a string assembly name such as `mm10` or `hg38`
-o, –output	Output folder path where results will be stored. Default is current working directory.
–barlabel	x-axis label of boxplot if –htype=tribar. Default is `Set size`
–barcolor	Boxplot color (hex vlaue or name, e.g. blue). Default is `#53cfff`.
–fontsize	Label font size. Default is `8`.
–title	Heatmap main title. Default is `Pairwise intersection`
–space	White space between barplt and heatmap, if –htype=tribar. Default is `1.3`.
–figtype	{pdf,svg,ps,tiff,png} Figure type for the plot. e.g. –figtype svg. Default is `pdf`
–figsize	Figure size for the output plot (width,height). e.g. –figsize 8 8
–dpi	Dots-per-inch (DPI) for the output. Default is: `300`.
–scriptonly	Set to generate Rscript only, if R/Corrplot package is not installed. Default is `False`
–test	This will run the program on test data.

Note: The option `--htype=tribar` will generate a horizontal bar plot with an adjacent heatmap rotated 45 degrees to show the lower triangle of the matrix comparing all sets of bars. If you want to view upper triangle, please `--triangle upper`. It's only recomended to use `tribar` if `compute` is set to `jaccard` or `fisher`.

CHAPTER 5

Example gallery

Here we provide some examples of how Intervene can be used to generate different types of set intersection plots.

Venn module examples

In this example, a 3-way Venn diagram of ChIP-seq peaks of histone modifications (H3K27ac, H3Kme3 and H3K27me3) in hESC from ENCODE data (Dunham et al., 2012).

```
intervene venn -i ~/ENCODE/data/H3K27ac.bed ~/ENCODE/data/H3Kme3.bed ~/ENCODE/data/
↪H3K27me3.bed --filenames
```

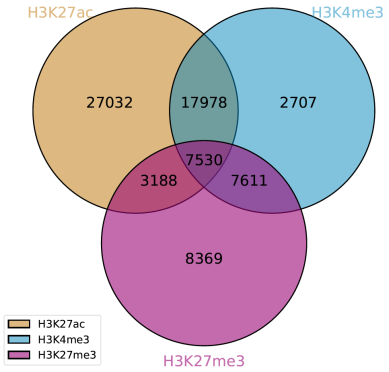

By adding one more BED file to `-i` argument, Intervene will generate a 4-way Venn diagram of overlap of ChIP-seq peaks.

```
intervene venn -i ~/ENCODE/data/H3K27ac.bed ~/ENCODE/data/H3Kme3.bed ~/ENCODE/data/
↪H3K27me3.bed  ~/ENCODE/data/H3Kme2.bed --filenames
```

Read more about the `venn` diagrams module here:

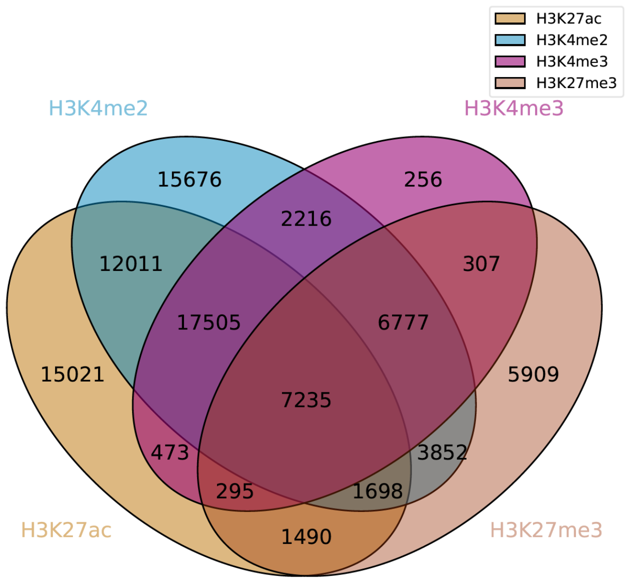


```
intervene venn --help
```

UpSet module examples

In this example, a UpSet plot of ChIP-seq peaks of four histone modifications (H3K27ac, H3Kme3 H3Kme2, and H3K27me3) in hESC from ENCODE data (Dunham et al., 2012).

```
intervene upset -i ~/ENCODE/data/H3K27ac.bed ~/ENCODE/data/H3Kme3.bed ~/ENCODE/data/
↪H3K27me3.bed ~/ENCODE/data/H3Kme2.bed --filenames
```

Read more about the `upset` module:

```
intervene upset --help
```

Pairwise module examples

In this example, we performed a pairwise intersections of super-enhancers in 24 mouse cell and tissue types from dbSUPER (Khan and Zhang, 2016) and showed the fraction of overlap in heatmap.

```
intervene pairwise -i ~/dbSUPER/mm9/*.bed --filenames --compute frac --htype pie
```

By setting the `--htype` to `color` will produce this plot.

```
intervene pairwise -i ~/dbSUPER/mm9/*.bed --filenames --compute frac --htype color
```

By setting the `--htype` to `tribar` will produce a triangular heatmap and with a bar-plot of set sizes.

```
intervene pairwise -i ~/dbSUPER/mm9/*.bed --filenames --compute frac --htype tribar
```

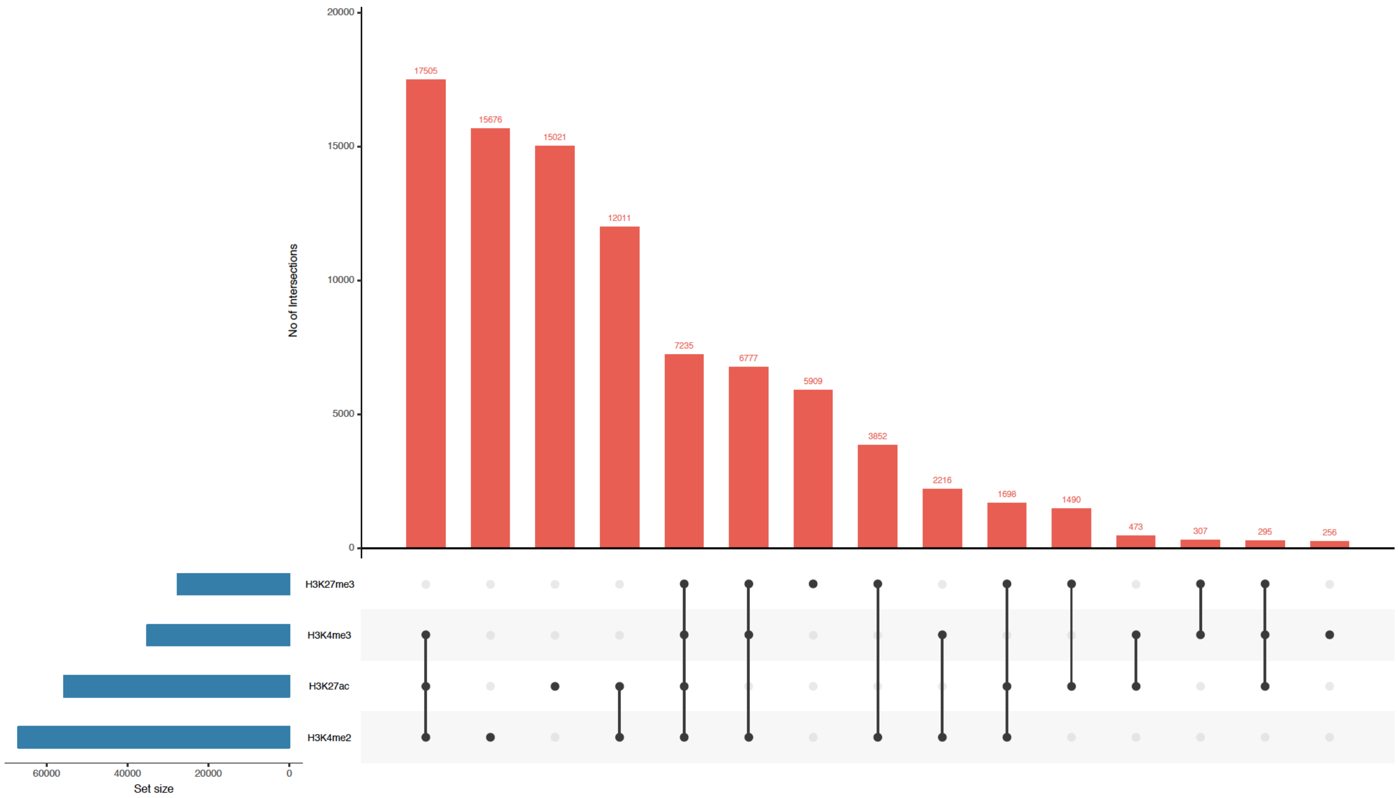

Note: Please make sure that the `tribar` will only show lower triangle of the matrix as heatmap and diagoals are set to zero. It recommended to use this if `--compute is set to ``jaccard`, `fisher` or `reldist`.

Read more about the `pairwise` module here:

```
intervene pairwise --help
```

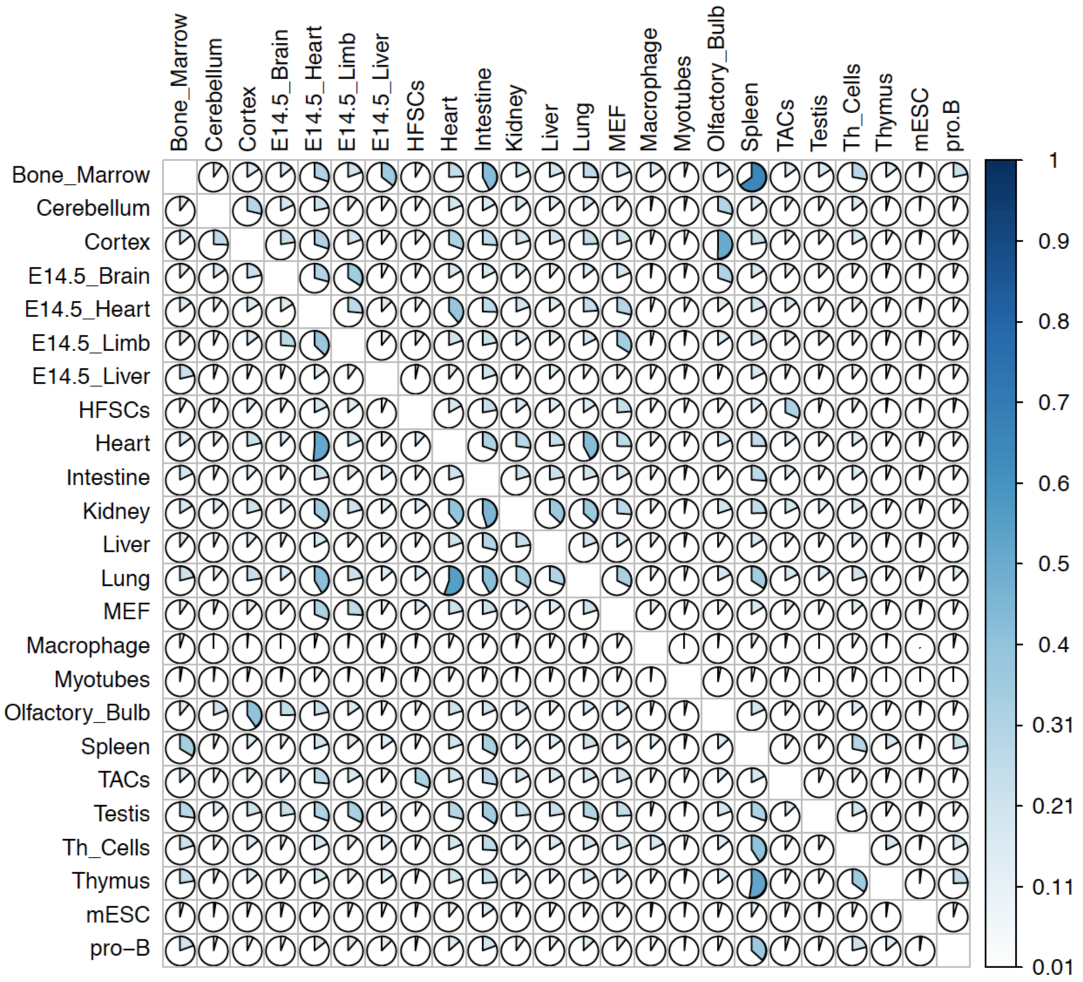
Bone_Marrow
Cerebellum
Cortex
E14.5_Brain
E14.5_Heart
E14.5_Limb
E14.5_Liver
HFSCs
Heart
Intestine
Kidney
Liver
Lung
MEF
Macrophage
Myotubes
Olfactory_Bulb
Spleen
TACs
Testis
Th_Cells
Thymus
mESC
pro.B
Bone_Marrow
Cerebellum
Cortex
E14.5_Brain
E14.5_Heart
E14.5_Limb
E14.5_Liver
HFSCs
Heart
Intestine
Kidney
Liver
Lung
MEF
Macrophage
Myotubes
Olfactory_Bulb
Spleen
TACs
Testis
Th_Cells
Thymus
mESC
pro–B
1
0.9
0.8
0.7
0.6
0.5
0.4
0.31
0.21
0.11
0.01

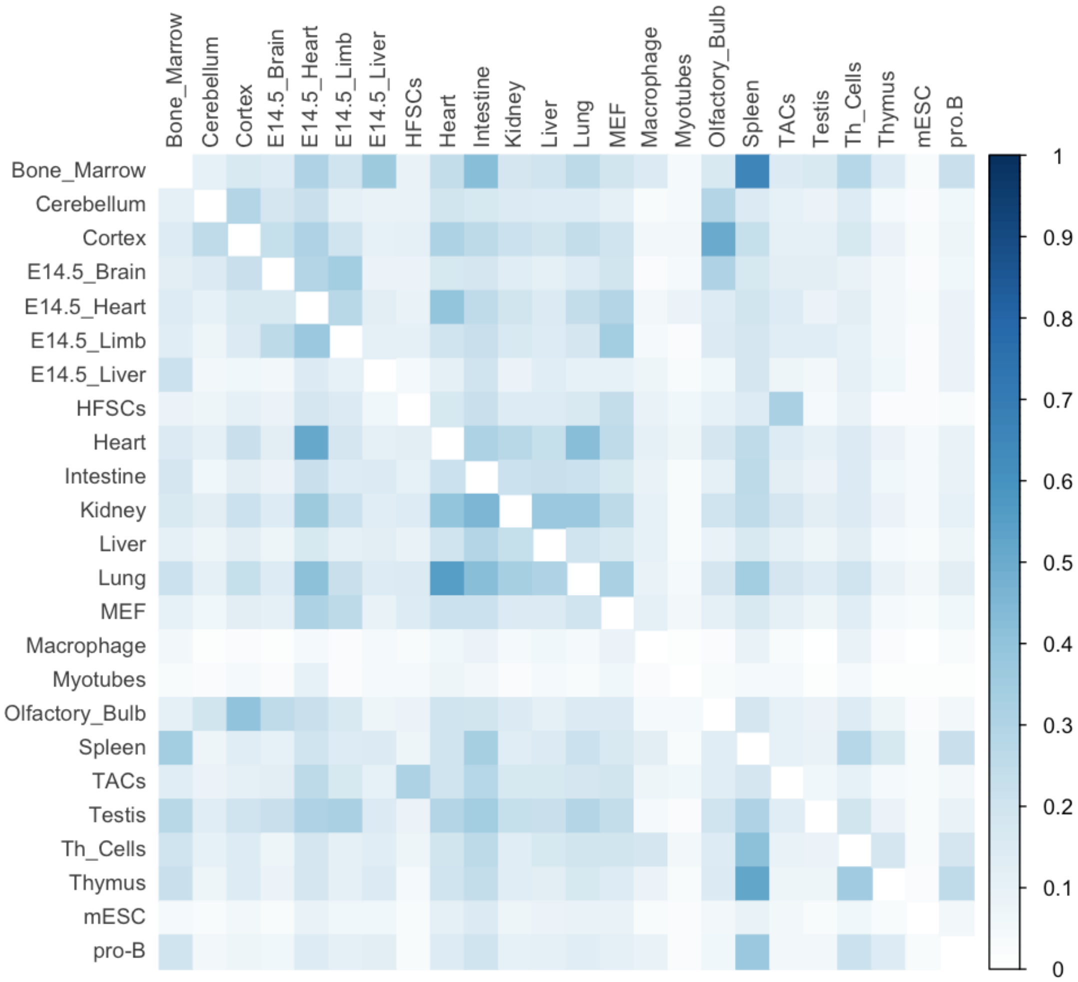
Bone_Marrow
Cerebellum
Cortex
E14.5_Brain
E14.5_Heart
E14.5_Limb
E14.5_Liver
HFSCs
Heart
Intestine
Kidney
Liver
Lung
MEF
Macrophage
Myotubes
Olfactory_Bulb
Spleen
TACs
Testis
Th_Cells
Thymus
mESC
pro.B
pro-B
1
0.9
0.8
0.7
0.6
0.5
0.4
0.3
0.2
0.1
0

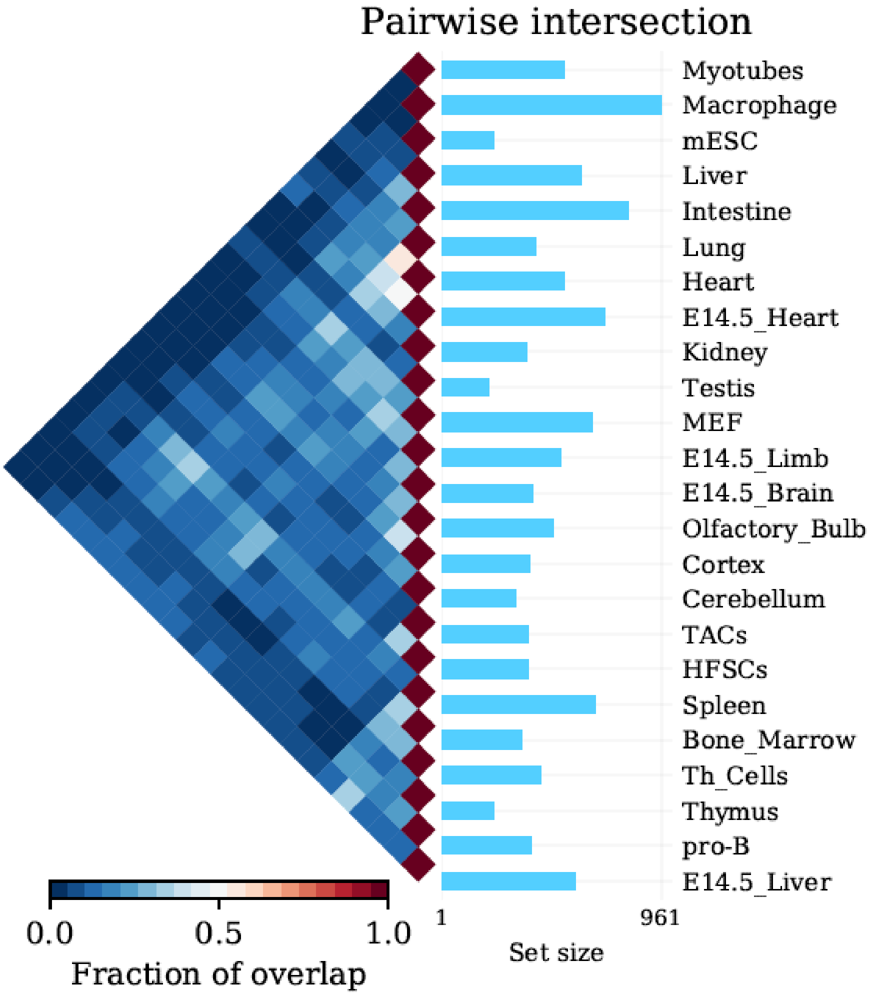
Pairwise intersection
Myotubes
Macrophage
mESC
Liver
Intestine
Lung
Heart
E14.5_Heart
Kidney
Testis
MEF
E14.5_Limb
E14.5_Brain
Olfactory_Bulb
Cortex
Cerebellum
TACs
HFSCs
Spleen
Bone_Marrow
Th_Cells
Thymus
pro-B
E14.5_Liver
0.0
0.5
1.0
Fraction of overlap
1
961
Set size

CHAPTER 6

Interactive Shiny App

Introduction

Intervene Shiny App provides an interactive interface for intersection and effective visualization of gene or genomic region sets. Currently, Shiny app does not acccept genomic regions as input, but the text files generated by Interve's command line interface can be easily uploaded to further explore and customize the plots in an interactive way. Intervene has three modules: venn to generate Venn diagrams of up-to 6 sets, upset to generate UpSet plots of more than 3 sets and pairwise to compute and visualize pariwise intersections as clustered heatmap.

Venn module

Intervene's venn module provides up-to 6-way classical, Chow-Ruskey and Edwards' Euler/Venn diagrams to visualize the intersections of genomic regions or lists.

Usage instructions

To use this venn module, you can upload a correctly formatted csv/text file, with lists of names. Each column represents a set, and each row represents an element (names/gene/SNPs).

Before uploading the file, choose the correct separator, wheather the names in each column are seperated by a ' , ' choose comma, by a ' ; ' choose semicolon, or by tabs choose tab.

Header names (first row) will be used as set names.

Intervene uses the Vennerable R package to generate different Venn diagrams.

UpSet module

Intervene's UpSet modules can be used to visualize the intersection of multiple genomic region sets using UpSet plots.

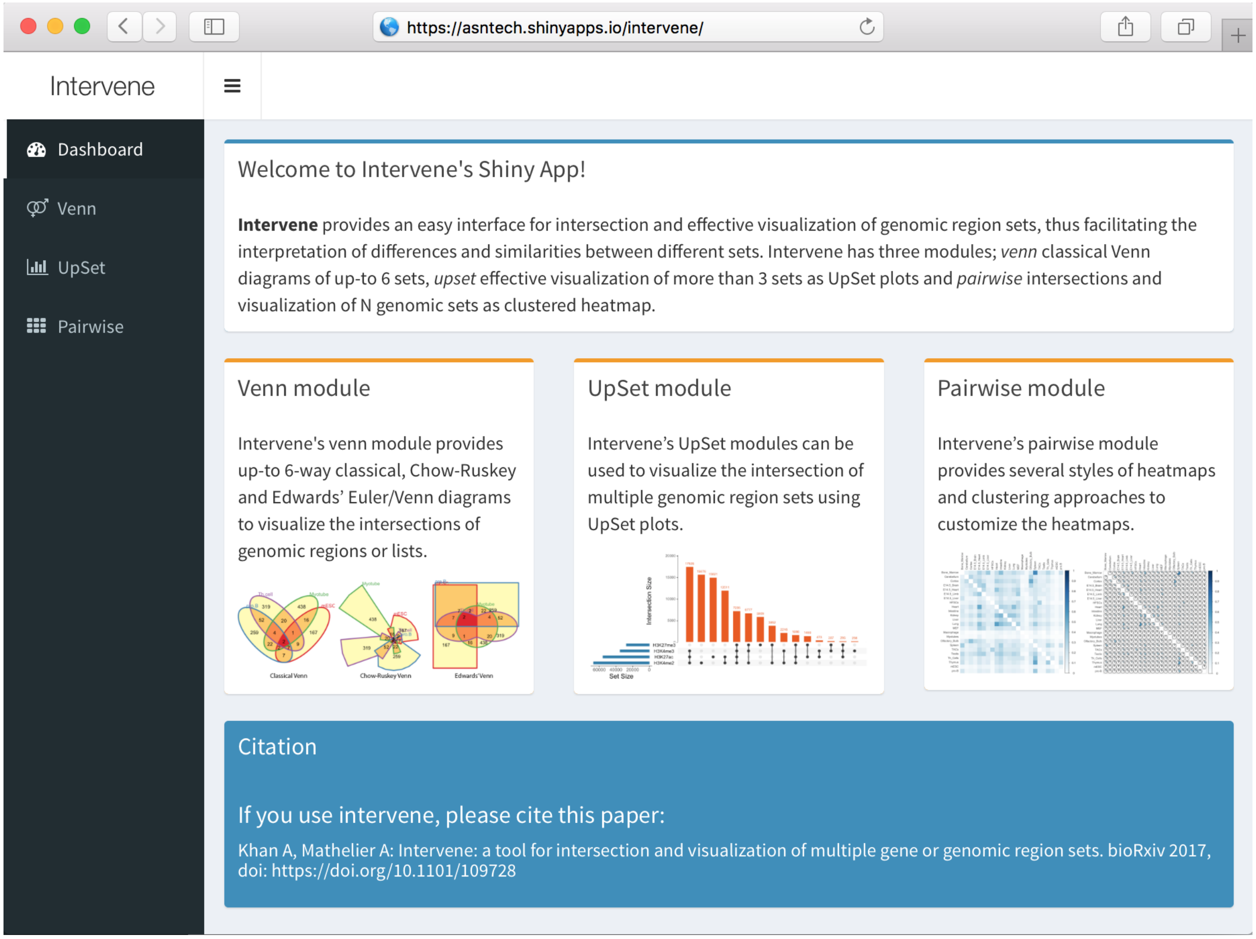
https://asntech.shinyapps.io/intervene/
Intervene
Dashboard
Venn
UpSet
Pairwise
Welcome to Intervene's Shiny App!
Intervene provides an easy interface for intersection and effective visualization of genomic region sets, thus facilitating the interpretation of differences and similarities between different sets. Intervene has three modules; venn classical Venn diagrams of up-to 6 sets, upset effective visualization of more than 3 sets as UpSet plots and pairwise intersections and visualization of N genomic sets as clustered heatmap.
Venn module
Intervene's venn module provides up-to 6-way classical, Chow-Ruskey and Edwards' Euler/Venn diagrams to visualize the intersections of genomic regions or lists.
UpSet module
Intervene's UpSet modules can be used to visualize the intersection of multiple genomic region sets using UpSet plots.
Pairwise module
Intervene's pairwise module provides several styles of heatmaps and clustering approaches to customize the heatmaps.
Citation
If you use intervene, please cite this paper:
Khan A, Mathelier A: Intervene: a tool for intersection and visualization of multiple gene or genomic region sets. bioRxiv 2017, doi: https://doi.org/10.1101/109728

Usage instructions

To use this module you can upload a correctly formatted .csv or text file, encoded in binary. Before uploading the file, choose the correct separator, wheather the names in each column are seperated by a ' , ' choose comma, by a ' ; ' choose semicolon, or by tabs choose tab. Header names (first row) will be used as set names.

UpSet module takes three types of inputs.

List type data

List data is a correctly formatted csv/text file, with lists of names. Each column represents a set, and each row represents an element (names/gene/SNPs). Header names (first row) will be used as set names.

Binary type data

In the binary input file each column represents a set, and each row represents an element. If a names is in the set then it is represented as a 1, else it is represented as a 0.

Combination/expression type data

Combination/expression type data is the possible combinations of set intersections. User can copy/past the combinations of intersection from the Intervene commnad line interface. For example;

H3K4me2&H3K4me3=2216, H3K4me2&H3K4me3&H3K27me3=6777, H3K27me3=5909, H3K4me3&H3K27me3=307, H3K4me3=256, H3K4me2&H3K27me3=3852, H3K4me2=15676, H3K27ac&H3K4me2&H3K4me3&H3K27me3=7235, H3K27ac&H3K4me2&H3K4me3=17505, H3K27ac&H3K4me2=12011, H3K27ac&H3K4me2&H3K27me3=1698, H3K27ac&H3K4me3=473, H3K27ac&H3K4me3&H3K27me3=295, H3K27ac&H3K27me3=1490, H3K27ac=15021

Intervene uses the UpSetR R package for visualization.

Pairwise module

Intervene's pairwise module provides several styles of heatmaps and clustering approaches to customize the heatmaps.

Usage instructions

To use pairwise module, you can upload a pairwise matrix file in .csv/txt format. Each column and row represents pairwise fraction of overlap/count etc between different names/genomic region sets.

Before uploading the file, choose the correct separator, wheather the matrix file is seperated by a ' , ' choose comma, by a ' ; ' choose semicolon, or by tabs choose tab.

Pairwise module takes input of two types:

List type data

List data is a correctly formatted csv/text file, with lists of names. Each column represents a set, and each row represents an element (names/gene/SNPs). Header names (first row) will be used as set names.

Pairwise matrix data

A pairwise matrix type data is a matrix of size NxN (all pairwise combinations) with values as number/fraction of overlap between two corresponding sets. For genomic region sets user can use the commpnad line interface of Intervene and upload the generated matrix here as matrix type.

For example here is the demo data generated by Intervene's command line interfacce for super-enhancers(SEs) of different cell/tissue-types from dbSUPER.

Intervene uses the Corrplot and plotly R packages to plot heatmap

Availability

The Intervene Shiny App is freely available at https://asntech.shinyapps.io/intervene

CHAPTER 7

Support

If you have questions, or found any bug in the program, please write to us at `aziz.khan[at]ncmm.uio.no` and `anthony.mathelier[at]ncmm.uio.no`.

CHAPTER 8

Citation

If you use plots or any results obtained from the Intervene tool, please cite:

- *Khan A, Mathelier A: Intervene: a tool for intersection and visualization of multiple gene or genomic region sets. bioRxiv 2017, doi: https://doi.org/10.1101/109728.
